# Performance of Family Health Strategy Nurses in LGBT+ Healthcare

**DOI:** 10.1590/0034-7167-2022-0514

**Published:** 2023-10-09

**Authors:** Ariane Tufaile Paiva, Flávio Adriano Borges, Pablo Ramon Carloni, Walkiria Jéssica Araújo Silveira, Márcia Niituma Ogata, Maria do Perpétuo Socorro de Sousa Nóbrega, Luciano Garcia Lourenção, Guadalupe Calvo García

**Affiliations:** I Universidade Federal de São Carlos. São Carlos, São Paulo, Brazil; II Universidade de São Paulo. São Paulo, São Paulo, Brazil; III Universidade Federal do Rio de Janeiro. Rio de Janeiro, Rio de Janeiro, Brazil; IV Universidade de Cádiz. Puerto Real, Spain

**Keywords:** Nursing, Primary Health Care, Public Health, Sexual and Gender Minorities, Sexuality., Enfermería, Primeros Auxilios, Salud Publica, Minorías Sexuales y de Género, Sexualidad., Enfermagem, Atenção Primária à, Saúde, Saúde Pública, Minorias Sexuais e de Gênero, Sexualidade.

## Abstract

**Objective:**

To analyze the role of Family Health Strategy (FHS) nurses in the health care of LGBT+ individuals.

**Methods:**

This qualitative study is based on Institutional Analysis. Data was collected in August 2021 through semi-structured interviews with 14 Family Health Strategy nurses from municipalities in the state of São Paulo. The data was processed using the IRaMuTeQ® software and analyzed lexically.

**Results:**

The textual corpus gave rise to three themes, which addressed the nurses’ practice, the difficulties and challenges they face in providing care to LGBT+ individuals, and the direct association of LGBT+ individuals with sexually transmitted infections.

**Conclusion:**

Lack of preparedness, access to information, and the need for expanded listening skills are still gaps in the performance of FHS nurses in caring for LGBT+ individuals. However, fostering acceptance and building strong relationships have been effective strategies in bridging the gap in nursing care for the LGBT+ community.

## RESUMO

**Objetivo:**

Analisar a atuação de enfermeiras da Estratégia Saúde da Família na atenção à saúde de pessoas LGBT+.

**Métodos:**

Estudo qualitativo baseado na Análise Institucional. Os dados foram coletados em agosto de 2021 por meio de entrevistas semiestruturadas realizadas com 14 enfermeiras da Estratégia Saúde da Família em municípios do Estado de São Paulo. Foram processados pelo software IRaMuTeQ® e analisados lexicalmente.

**Resultados:**

O corpus textual gerou três temas, que abordam a prática das enfermeiras, as dificuldades e desafios enfrentados por elas no atendimento às pessoas LGBT+ e a associação direta de pessoas LGBT+ às infecções sexualmente transmissíveis.

**Considerações finais:**

O despreparo, o acesso à informação e o desenvolvimento de uma escuta ampliada ainda são lacunas na atuação de enfermeiras da Estratégia Saúde da Família no cuidado com pessoas LGBT+, mas o acolhimento e a criação de vínculos têm sido estratégias adotadas para aproximar a assistência de enfermagem das pessoas LGBT+.

## RESUMEN

**Objetivo:**

Analizar la actuación de enfermeras de la Estrategia Salud de la Familia en la atención a la salud de personas LGBT+.

**Métodos:**

Estudio cualitativo basado en el Análisis Institucional. Los datos fueron recolectados en agosto de 2021 a través de entrevistas semiestructuradas realizadas con 14 enfermeras de la Estrategia Salud de la Familia en municipios del Estado de São Paulo. Fueron procesados por el software IRaMuTeQ® y analizados lexicalmente.

**Resultados:**

El corpus textual generó tres temas, que abordan la práctica de las enfermeras, las dificultades y desafíos enfrentados por ellas en la atención a las personas LGBT+ y la asociación directa de personas LGBT+ con las infecciones de transmisión sexual.

**Conclusiones:**

La falta de preparación, el acceso a la información y el desarrollo de una escucha ampliada aún son lagunas en la actuación de enfermeras de la Estrategia Salud de la Familia en el cuidado con personas LGBT+, pero el acogimiento y la creación de vínculos han sido estrategias adoptadas para acercar la asistencia de enfermería a las personas LGBT+.

## INTRODUCTION

In Brazil, Primary Health Care (PHC) is considered the preferred gateway to the public health system and comprises a set of individual, collective/community health actions that involve promotion, prevention, diagnosis, treatment, rehabilitation, harm reduction, palliative care, and health surveillance, developed by a multidisciplinary team, including the nurse^(1)^.

The Family Health Strategy (FHS) is understood as the model for the development of PHC in the country^(1)^ and, according to the National Primary Care Policy (from portuguese *Política Nacional de Atenção Básica -* PNAB), the minimum FHS team must be composed of: a physician, preferably a specialist in family and community medicine; a nurse, preferably a specialist in family health; two nursing assistants and/or technicians and community health agents (CHA). In addition to these professionals, a dentist, preferably a specialist in family health, a dental health assistant or technician, and endemic disease control agents (EDC) may also be part of the team^(1)^.

The FHS nurse is responsible for carrying out and supervising user reception, and is almost always the first professional of higher level that users have contact with when they arrive at the Family Health Unit (FHU). In addition, these professionals should base their functions on public health policies and the principles of the Unified Health System (from portuguese *Sistema Único de Saúde* - SUS), which do not allow exclusions of any nature, in addition to the obligation to promote equity in health care^(2)^.

In the pursuit of guaranteeing these principles, in 2011 the Brazilian National Policy for Comprehensive Health Care of Lesbians, Gays, Bisexuals, Transvestites and Transsexuals (PNLGBT) was created with the aim of reinforcing the aforementioned, with the objective of promoting the integral health of this population, eliminating institutional discrimination, prejudice, reducing inequalities, and consolidating Unified Health System as a universal, integral, and equitable system^(3)^.

In this context, the relevance of attention to the health of lesbians, gays, bisexuals, transvestites, transsexuals, transgender people, and other dissident sexual orientations and gender identities from the hetero-cis-normative and binary model (LGBT+) is identified. For clarification, this study uses the acronym LGBT+ based on PNLGBT itself, which explicitly mentions only the LGBT population. However, it is understood to be an acronym that carries diversity and is in a continuous process of transformation and, consequently, new compositions and modifications.

LGBT+ individuals face discriminatory barriers in family, work, and school environments, as well as in healthcare settings^(4)^. According to the National Alliance on Mental Illness office^(5)^, one in five LGBT+ individuals do not disclose their sexual orientation to healthcare professionals during treatment. Due to fear of discrimination by healthcare professionals, transgender individuals tend to avoid seeking healthcare services, even when ill, or abandon proposed treatment^(6)^.

Brazilian studies^(7-8)^ have found that access to healthcare services for the LGBT+ population is fraught with embarrassment and prejudice, highlighting exclusion, neglect, omission, and indifference as the main feelings expressed by these individuals, in addition to how their health issues are neglected in the context of FHS team work^(4)^. Numerous adversities faced by professionals in caring for the specific needs of LGBT+ individuals are known, such as difficulties in conducting comprehensive listening and recognizing the real demands of this population^(4,6)^; disarticulation of the healthcare network in favor of LGBT+ care^(9-10)^; training deficit for meeting the needs of this population^(2)^, in addition to the lack of permanent health education spaces for these professionals^(10-11)^.

It is clear that the situation experienced by the LGBT+ population under the social determination of the health process, where required health needs are not met, leads to increased risks and losses, placing these individuals in a vulnerable condition^(4)^. Therefore, it is considered relevant to provide visibility and legitimacy to the space of reception and care for the LGBT+ population by nurses working in the FHS.

In this context, the present study is the result of a loco-regional research that made it possible to reach a broader territory of a Brazilian state, which contributes to advancing knowledge in the area and provides opportunities for directing paths towards the development of public policies that mitigate the deficits presented by nurses in guaranteeing SUS principles in their daily practices. In addition, they support the development of an accurate diagnosis capable of grounding new interventional research that seeks to transform nurses’ daily practices through the development of Permanent Health Education (PHE).

## OBJECTIVE

To analyze the performance of nurses in the Family Health Strategy in the healthcare of LGBT+ people.

## METHODS

### Ethical aspects

The project that gave rise to this study was approved by the Research Ethics Committee under number 4,647,705 in 2021. The research was conducted in accordance with resolution 466/12^(12)^, with the use of informed consent forms signed by all research participants, as required by current laws in the country. To preserve anonymity and in compliance with ethical resolutions that support the development of research with human subjects in the country, the interviewed professionals were identified by the acronym ENF, followed by the corresponding cardinal number.

### Theoretical-methodological framework

The study used the theoretical framework of Institutional Analysis (IA), which originated from the institutionalist movement of 1960s France and was introduced in Brazil in the 1970s. IA aims to capture a specific social and organizational reality, and is epistemologically centered on dialectical theory, critical analysis of Freudian psychoanalysis, and other theoretical constructs^(13-14)^.

This is an interdisciplinary theoretical framework that is applicable based on the understanding of professional practice as a scenario for the construction of the worker, who also recognizes themselves through looking and being with others, while also being constituted from the other^(13)^. Thus, it is understood that this theoretical framework contributed to the analysis of the data in this study by being grounded in principles that articulate a perspective of finding answers based on what is not always said or made explicit in the speech, gestures, and explicit statements made by people.

To accomplish this, some concepts of IA were used to analyze the healthcare of LGBT+ people in the context of the work of nurses in the FHS. It is important to note that these concepts are used to direct analytical clues for intervention processes in IA, and not as a checklist to be followed by every study that uses this theoretical and methodological framework. These concepts were: institution (socially established norms and rules), instituted (what is visible in the institution), instituting (what moves and provokes the instituted), institutionalization (dialectical relationship between the instituted and the instituting), and analyzer (what reveals and makes the institution evident)=^(13-14)^.

### Study Type

This is a descriptive, qualitative study based on the theoretical constructs of Institutional Analysis. The Consolidated Criteria for Reporting Qualitative Research (COREQ)^(15)^ were adopted as criteria.

### Data Source

The sample consisted of 14 nurses working in units of the FHS in municipalities in the state of São Paulo, Brazil. The inclusion criteria were being a nurse working in the FHS of any municipality in the state of São Paulo. The exclusion criterion was not having cared for LGBT+ individuals in their professional practice as an FHS nurse.

The participants were recruited non-intentionally through the dissemination of the research and a link to a form via digital media (Facebook, WhatsApp groups, Twitter, etc.). Professionals who voluntarily agreed to participate in the research had to fill out a form to enable contact by the researchers later. This process was hampered by the intense work scenario caused by the COVID-19 pandemic, but it did not compromise data collection.

The research was disseminated and responses to the form were accepted until sample saturation was reached. This means that the statements of the next nurses did not generate new information for the analytical context^(16)^, reflecting in quantity and intensity the dimensions of the phenomenon studied and the quality of actions and interactions developed during the research^(17)^. Thus, the researchers adopted the following steps to verify sample saturation: a) transcription of interviews; b) immersion in each conducted interview; c) compilation of individual analyses of each interview; d) gathering of preliminary themes from the previous stage; e) confirmation of theoretical saturation for each preliminary theme; f) running the general textual corpus and elaboration of themes; g) visualization of saturation in the confluence of preliminary themes with the themes^(16)^.

### Data Collection and Organization

Data was collected in August 2021 through the completion of a form containing sociodemographic data such as gender, age, city of professional practice, length of time as a nurse, whether they had a specialization in FHS or PHC, and length of time working in this area of healthcare. Participants were also asked to provide their name and phone number for further semi-structured individual interviews, which were conducted virtually using the Google Meet® digital platform. The guiding questions were presented and discussed beforehand among the researchers in order to ensure their quality before implementation.

The following guiding questions were used in the study: a) Have you provided care to lesbians, gays, bisexuals, transgender, or other individuals who do not identify as heterosexual? b) What do you consider important to address in healthcare services for the LGBT+ population? c) What are the facilitators and barriers you encounter when providing healthcare services to the LGBT+ population? The duration of each interview was approximately 30 minutes. The interviews were recorded in MP4 format with participants’ permission and later transcribed and analyzed.

### Data Analysis

The data was analyzed using lexical analysis, which sought to identify themes of significance based on coding units present in the participants’ reports^(18)^. For this purpose, the interview transcriptions were compiled into textual corpora and processed using the software “*Interface de R pour les Analyses Multidimensionnelles de Textes et de Questionnaires*” (IRAMUTEQ®) and subsequently analyzed.

IRAMUTEQ® performs statistical analysis by grouping words with semantic similarity present in the textual corpus. The corpus is divided by the software into text segments (TS), which consist of textual fragments that preserve semantic proximity to each other ^(19)^.

Thus, the textual corpus was prepared and revised to eliminate typing errors and standardize acronyms and expressions (while preserving the same meanings). Adjectives, adverbs, nouns, verbs, and forms not recognized as word categories were included in the software analysis process. Next, Classical Textual Statistics and Descending Hierarchical Classification (CHD) of the words present in the text segments were analyzed according to the value of the Chi-Square test (x2 > 3.80) in order to include those with statistically significant values (p<0.05).

After the lexical analysis, the findings were compared with the theoretical framework of Institutional Analysis, which is understood as an approach that uses revealing concepts of institutions based on interventions grounded in the analysis of practices and discourses of subjects^(13-14)^.

## RESULTS

The recruitment form was filled out by 22 FHS nurses, of which eight were excluded for not responding to attempts to schedule the interview. Therefore, 14 FHS nurses from 10 different municipalities in the state of São Paulo participated in the study, all female (which is why the authors chose to refer to the study participants in the feminine gender), aged between 20 and 40 years old, with more than 1 year of nursing education, all with a specialization in family health, and with a tenure in the FHS greater than one year, as presented in Table 1.

**Table 1:** Sociodemographic and professional characteristics of interviewed nurses, São Paulo, Brazil, 2021

Variables	n(%)
Sex	
Female	14(100)
Age range (Years)	
20-30	6 (42.8)
31-40	8 (57.2)
Professional training time	
< 1 year	-
1-3 years	3 (21.5)
3-5 years	2 (14.2)
5-10 years	6 (42.8)
> 10 years	3 (21.5)
Do you have a specialization in Family Health?	
Yes	14 (100)
No	-
Time of work in the Family Health Strategy (FHS)	
< 1 year	-
1-3 years	3 (21.5)
3-5 years	4 (28.5)
5-10 years	4 (28.5)
> 10 years	3 (21.5)

The overall corpus of interview transcripts consisted of 14 texts, divided into 215 TS, with 170 TS (79.06%) utilized. A total of 6,203 occurrences (words, forms or terms) emerged, with 802 distinct words and 691 with only one occurrence. The analyzed content was categorized into three themes, namely Theme 1 with 69 TS (41.0%), Theme 2 with 67 TS (40.0%), and Theme 3 with 31 TS (19.0%).

**Figure 1:**
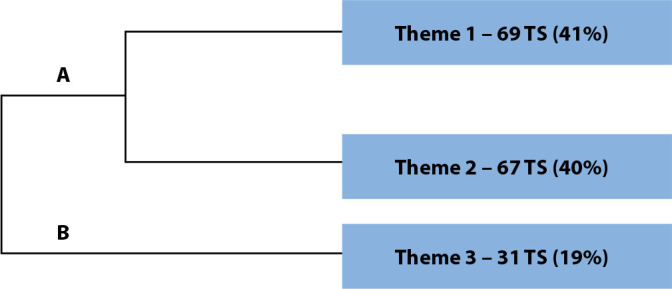
Dendrogram of the textual corpus analysis of the interviews, São Paulo, Brazil, 2021

It is worth noting that the three themes were divided into two branches (A and B) of the overall analysis corpus (Figure 1). Subcorpus A, “Experiences in caring for LGBT+ individuals”, contained the corresponding segments for Themes 1 (“Difficulties and challenges in caring for LGBT+ individuals”) and 2 (“Professional practices used in caring for LGBT+ individuals”). Subcorpus B, “Clinical Focus”, consisted of Theme 3 (“LGBT+ individuals and sexually transmitted infections”).

### Difficulties and challenges in providing care to LGBT+ individuals

This theme comprises 69 TS (41%) of the total corpus analyzed, consisting of words such as “situation” (x^2^ = 12.1), “respect” (x^2^ = 10.8), “difficulty” (x^2^ = 9.1), “knowledge” (x^2^ = 7.5), and “question” (x^2^ = 4.8).

As demonstrated by the speech fragments, it is possible to identify the nurses’ perception of the difficulties faced by LGBT+ individuals; the unpreparedness of healthcare professionals to deal with this population, both in terms of access to information and their own university education; and the difficulty in developing an attentive, sensitive, and capable listening to identify the real health needs presented by LGBT+ individuals.

*Because until today we see that there is a problem with the difficulty of welcoming this population group. Nursing is usually not trained. I’m not generalizing, but the nursing staff is not trained to provide this care.* (N1)

*I think that a lot of information is still not widely disseminated.* (N5)

*There is still taboo, discrimination. So, there is this unpreparedness of nursing for specific health issues of LGBT people.* (N9)

*So I think that sometimes nursing professionals are not trained to recognize the multidimensionality of individuals, of LGBT people.* (N4)

### Professional practices used in the care of LGBT+ individuals

This theme comprises 67 TS (40%) of the total corpus analyzed. It is composed of words such as “serve” (x^2^ = 18.8); “taboo” (x^2^ = 14.9); “prejudice” (x^2^ = 12.1); “bond” (x^2^ = 10.9); “transsexual” (x^2^ = 7.4).

The analysis identifies that nurses perceive some aspects that interfere with the care of LGBT+ individuals and may compromise the effectiveness of health care. The influence of the professional’s view in the provision of care and the need for adequate reception are fundamental to the care of the LGBT+ population, as these individuals already arrive at health services stigmatized due to discriminatory situations they experience on a daily basis. In addition, some strategies have been identified by nurses and have been used in the provision of care for LGBT+ individuals, such as reflecting on reception and seeking to establish a bond; respect and adoption of strategies that generate security in the LGBT+ user, promoting their adherence to the care provided.

*Individuals who are homophobic end up bringing their experiences and beliefs to the care they provide, and end up assaulting and violating other people, even if unintentionally or indirectly. And yes, I believe it is a lack of respect in the care provided.* (N2)

*It is a population that usually does not seek health services, especially transgender individuals.* (N3)

*If you are a person who welcomes them when they enter, they will certainly not have such a big barrier to speak with you. For me, professional attitude weighs heavily.* (N10)

*I think that when you have a bond with the person, you get to know them, all of this helps in the care not only of this population, but everyone. I think respect is also important.* (N7)

### People from the LGBT+ community and sexually transmitted infections

This theme comprises 31TS (19%) of the total corpus analyzed, consisting of words such as “reproductive” (x^2^ = 27.7); “couple” (x^2^ = 25.4); “sexually transmitted infection” (x^2^ = 21.4); “Pap smear” (x^2^ = 20.1); “sexual” (x^2^ = 19.3).

It was possible to identify that nurses still link LGBT+ individuals to sexually transmitted infections (STIs) and therefore consider it relevant to address this issue during clinical care. Additionally, there is a specific focus on the female population, namely, lesbians.

*Many still, I perceive, do not take preventive measures when it comes to sexually transmitted infections. Most of the gay men who seek care come in for rapid testing.* (N13)

*Regarding sexual and reproductive health, we have, for example, the consultation for Pap smear collection.* (N6)

*I think biological issues always come up, but prevention of sexually transmitted infections becomes important again.* (N11)

*In relation to lesbians, I notice that in consultations, it’s always one couple of lesbians, the one who identifies as feminine who seeks care for their sexual and reproductive health. The one who sees herself as more masculine does not.* (N14)

*Women, for instance, menstruate, get pregnant, have children and need to have their Pap smear test done annually, regardless of anything else.* (N8)

## DISCUSSION

Access to information is a sensitive factor in the formation process of any professional. For nursing and other health-related professions, the development of classes and practical experiences that provide an effective encounter between students, teachers, health professionals, and the community is essential for the development of knowledge, skills, and attitudes^(20)^.

Regarding the education of nurses for the health care of LGBT+ people and considering sexuality as an institution (due to its socially constructed norms and rules), the instituted perspective of compulsory and binary heteronormativity in directing what is outlined in relation to this institution is noteworthy, naturalizing this process. An example of this is the fact that the approach to health care for gay men is still related to STIs and for lesbian women to sexual and reproductive issues^(21)^, disregarding the rights that LGBT+ individuals have in accessing care for reproductive and fertility issues, leaving no doubt about the constant stigmas they face when needing to undergo these processes.

This is a non-compliance with the principle of comprehensiveness as a doctrinal principle of the SUS, which requires an expanded view, beyond acute conditions or what is institutionally established and operates in directing health practices. In this direction, primary health care professionals still face the comprehensiveness of care as something of great complexity to operationalize due to the diversity of values, desires, and expectations constructed in different life conditions in the territories^(22)^.

Because sexuality as an institution has an interdisciplinary character in its composition, encompassing biological, psychosocial, and cultural aspects, it requires formative approaches that awaken the articulation between knowledge, as occurs in the development of multiprofessional work, in teamwork, and in interprofessional and collaborative education^(23)^.

Several national and international studies have pointed out the shortage of health curricula that effectively address the theme of LGBT+ health care^(24-27)^. What is known is that when this theme appears in health education, it tends to be concentrated in extracurricular activities such as those proposed by student movements, extension activities, or research projects^(25)^. This phenomenon represents the institutionalization of sexuality within the realm of health professional education. It exists between traditional approaches, which remain rooted in outdated perspectives and the cis-hetero-normative-binary model of teaching sexuality, and progressive forces that aim to expand curricula, advocating for a more inclusive, flexible, and contemporary perspective.

Thus, there is a need to trigger instituting forces capable of provoking processes of curricular restructuring and reformulation in nursing undergraduate courses, seeking to ensure spaces that discuss gender (beyond the binary perspective: male and female), sexual orientation (expanding beyond the established heteronormative standard), and sexuality, in general. Such forces would act to broaden the professional’s perspective on caring for diversities, mitigating social inequalities, and especially paying greater attention to the health of LGBT+ people^(28)^.

Health education needs to be directly articulated to daily life, which, in turn, induces the production of technical-scientific knowledge^(23)^. Thus, ensuring spaces that favor the exchange of information among health professionals, sharing experiences, and continuing education are necessary conditions for the development of quality care practices^(23,29)^. Moreover, they are agendas capable of generating instituting forces in favor of transforming health work.

Both nurses and other health professionals in the FHS need to be in continuous contact with scientific knowledge, seeking to improve techniques, update protocols, modify approaches, and, not least, problematize their professional practice. It was with this in mind that the National Policy for Continuing Education in Health was created, with a view to incorporating critical reflection by health professionals on their work processes, aimed at discovering solutions to the daily problems of services^(4)^.

However, when we bring this discussion closer to the context of PHC, it is possible to identify a certain emptiness and little participation of health workers in the protected spaces intended for problematizing daily professional practice^(30)^. This emptiness constitutes an analyzer that highlights the praxis of health work, executed from a technical perspective and little reflection. Consequently, there is a decrease in the quality of care provided to the population. An example of this could be identified by the nurse’s view influencing the provision of care, something that could be problematized in certain spaces guaranteed within the daily work.

In this case, specifically, it also consists of not fulfilling the doctrinal principle of equity, which proposes to meet the various health needs presented in the territories differently. Health professionals who work in PHC also face difficulties in implementing this principle in the daily practice of health services due to the assistance limits (medical-centered, low listening, etc.) and structural (slowness of operational systems, worker deficits, etc.) experienced by them^(31)^.

he emptying of spaces for discussion and problematization of professional practice can occur due to the scarcity of human resources in PHC services, which hinders the realization of formative moments due to the work overload placed on professionals and the low valorization of these discussion spaces, such as team meetings, by municipal management^(30)^. That is, highlighting the analyzers “absence” and “non-existence of guaranteed spaces for the development of EPS” can generate instituting forces capable of transforming the instituted aspects of professional health practices, leading to a process of institutionalization that provides greater reflection on the act of health care delivery.

The concepts of “empathy” and “bond” themselves are subject to discussion and construction, also operating as possible analyzers capable of highlighting the institutionalization of professional practices in health. As strategies adopted by the nurses in this study, empathy and bond point towards the pursuit of developing a humane practice that preserves, respects, and guarantees the fundamental right to health of LGBT+ individuals^(32)^. However, understanding the needs of the LGBT+ population is necessary for the construction of knowledge and practices that underpin nursing care^(33-34)^, once again pointing to the relevance of a formative process regarding LGBT+ health.

The pursuit of strategies that make LGBT+ individuals feel comfortable enough to express themselves is highly relevant and constitutes an instituting force in clinical health. By recognizing LGBT+ individuals as human beings with rights and specific health needs that can only be expressed if they feel welcomed, these strategies contribute to the creation of a more inclusive healthcare environment^(35-36)^. However, it is widely recognized that barriers to accessing healthcare faced by the LGBT+ population often begin at the reception desk due to prejudiced and violent situations, such as the disrespect of their social names^(37-38)^.

This disrespect extends beyond the initial reception in FHU and is also observed in the clinical management of cases by healthcare professionals. Historically, healthcare for LGBT+ individuals has been primarily focused on the prevention of STIs, which has contributed to the direct association between LGBT+ sexual activity and promiscuity^(39)^. Moreover, FHS professionals’ social representations of LGBT+ individuals still carry an outdated connotation that links gender expression and identity with sexual orientation, such as the association of effeminate characteristics with gay men and masculinized characteristics with lesbian women^(10)^. This process must be transformed to achieve the institutionalization of clinical care in FHS, capable of considering gender diversity in various socio-cultural contexts, including the existence of agender individuals.

The findings of this study demonstrate the assistance provided by nurses to LGBT+ individuals within the context of the FHS, contributing to the identification of established practices revealed through the emergence of existing gaps in both university education and within the context of health work itself.

### Study Limitations

A limitation of this study is that it was developed only with female nurses. Although they make up the majority of the nursing workforce, it did not favor diversity of perspectives on the subject at hand.

### Contributions to Nursing, Health, or Public Policy

This study contributes to the SUS by addressing issues closely related to the work process within the context of PHC. It also contributes to the nursing field by shedding light on the difficulties faced by FHS nurses in providing healthcare to LGBT+ individuals and directing possible avenues capable of transforming this reality. The study aims to reduce inequalities and inequities in access to healthcare services, with an investment in university education, health education through the FHS, and the guarantee of basic principles that govern PHC as a first-contact and comprehensive service, centered on expanded listening and effective reception. Furthermore, this production induces discussions on the rights of LGBT+ individuals to access health services in the context of public policy.

## FINAL CONSIDERATIONS

The performance of FHS nurses in providing healthcare to LGBT+ individuals is still permeated by various difficulties and challenges, such as unpreparedness, limited access to information, and the need to develop an expanded listening approach that encompasses the health needs of LGBT+ individuals. This is reflected in the direct association of LGBT+ individuals with STIs and females with procreation, resulting in the provision of strictly gynecological services. However, some strategies have been adopted by FHS nurses with the aim of bringing nursing care closer to the LGBT+ population, such as fostering a welcoming environment and building relationships.
