# ERRATUM

**DOI:** 10.1590/0034-7167.20237605e05

**Published:** 2023-11-27

In the article “Performance of Family Health Strategy Nurses in LGBT+ Healthcare”, with DOI number: https://doi.org/10.1590/0034-7167-2022-0514, published in Revista Brasileira de Enfermagem, 2023;76(4): e20220514, authored:

Where it read:

**Walkiria Jéssica de Araújo Silveira^I^**

ORCID: 0000-0003-3144-8601

It reads:

**Walkiria Jéssica Araújo Silveira^I^**

ORCID: 0000-0003-3144-8601

